# Mechanism-based Classification of Pain for Physical Therapy Management in Palliative care: A Clinical Commentary

**DOI:** 10.4103/0973-1075.78458

**Published:** 2011

**Authors:** Senthil P Kumar, Sourov Saha

**Affiliations:** Department of Physiotherapy, Kasturba Medical College, Manipal University, Mangalore, India

**Keywords:** Mechanism-based classification, Pain rehabilitation, Pain sciences, Palliative physical therapy care

## Abstract

Pain relief is a major goal for palliative care in India so much that most palliative care interventions necessarily begin first with pain relief. Physical therapists play an important role in palliative care and they are regarded as highly proficient members of a multidisciplinary healthcare team towards management of chronic pain. Pain necessarily involves three different levels of classification–based upon pain symptoms, pain mechanisms and pain syndromes. Mechanism-based treatments are most likely to succeed compared to symptomatic treatments or diagnosis-based treatments. The objective of this clinical commentary is to update the physical therapists working in palliative care, on the mechanism-based classification of pain and its interpretation, with available therapeutic evidence for providing optimal patient care using physical therapy. The paper describes the evolution of mechanism-based classification of pain, the five mechanisms (central sensitization, peripheral neuropathic, nociceptive, sympathetically maintained pain and cognitive-affective) are explained with recent evidence for physical therapy treatments for each of the mechanisms.

## INTRODUCTION

Pain is the chief symptom reported by patients in a palliative care unit and most palliative care interventions begin first with pain relief.[1] Since pain is mainly measured using subjective reports of intensity and symptom behavior, it is often misinterpreted despite its popularity of being considered the fifth vital sign.[2]

Pain is a common complaint among patients seeking physical therapy services. Physical therapists play an important role in palliative care[3] and they are regarded as highly proficient members of a multidisciplinary healthcare team towards management of chronic pain.[4]

The effects of physical therapy in pain management extend far beyond the physical aspect of symptom control, into the behavioral domain of quality of life during therapeutic care.[5] Physical therapy comprises exercise therapy, electrotherapy, actinotherapy and manual therapy, all of which have been shown to be effective in a wide range of disease conditions such as neurological, musculoskeletal, psychiatric and end-of-life states in a palliative care setting.[3]

One of the initial steps in a physical therapist’s evaluation of patients with chronic pain is to identify the basis for symptoms and their occurrence.[6] Patients’ understanding and reporting of pain in terms of their symptoms and ‘pain experience’ influence therapists to provide physical therapy treatments in terms of diagnoses or syndromes. Whilst an adequate addressal of symptoms from disease states could be achieved upon understanding of pain mechanisms,[7] physical therapy treatment methods have their own mechanism-specific effects which when understood and applied, would lead to an effective adjunctive role in palliative symptom management.

We are witnessing a paradigm shift in pain medicine towards a mechanism-based approach[8] and in line with mechanism-based pharmacological treatments,[9] physical therapy treatments could be more effectively provided if such a mechanism-based approach is available.

Smart and Doody[10] found among physical therapists that there was a relatively lesser trend for the use of pain mechanisms in their clinical decision-making and clinical reasoning process. Later, Smart and Doody[11] evaluated the use of mechanism-based classification by expert musculoskeletal physiotherapists using qualitative methodology and found that therapists less often used cognitive-affective mechanisms in their clinical reasoning process of evaluation of pain in their patients. Deficiencies in knowledge about pain mechanisms would lead to potentially undesirable attitudes towards treating patients with chronic pain.[12]

In contrast to symptomatic and/or diagnosis-based treatments, mechanism-based treatments are most likely to succeed.[13] The objective of this clinical commentary is to update the physical therapists working in palliative care, on the mechanism-based classification of pain and its interpretation, with available therapeutic evidence for providing optimal patient care using physical therapy treatment options along such a mechanism-based approach.

## MECHANISM-BASED CLASSIFICATION OF PAIN

The works of various authors[7,14–16] inspired physical therapists like Mark Jones[17] to propose a mechanism-based clinical reasoning of pain. Now it is evident, however, that there are five mechanisms of pain:

Central sensitization/ central neurogenic mechanism/ central nociceptive mechanism

Peripheral sensitization/ peripheral neurogenic mechanism

Peripheral nociceptive mechanism

Sympathetically maintained pain/ sympathetically dependent pain mechanism

Cognitive-affective (psychosocial) mechanism

## MECHANISM-BASED CLASSIFICATION OF PAIN—FIVE MECHANISMS OF PAIN

### i. Central sensitization/central neurogenic mechanism

Central sensitization represents an enhancement in the function of neurons and circuits in nociceptive pathways caused by increases in membrane excitability and synaptic efficacy as well as by reduced inhibition and is a manifestation of the remarkable plasticity of the somatosensory nervous system in response to activity, inflammation, and neural injury.[18] Nijs *et al*.,[19] described the notable features of the presence of a central sensitization mechanism among patients with musculoskeletal pain: widespread hypersensitivity to bright light, touch, noise, pesticides, mechanical pressure, medications and high and low temperatures. Assessment of sensitivity and pressure pain thresholds at sites remote (anatomically unrelated) from the symptomatic site, during or after an activity and neural tissue provocation testing would elicit widespread symptom distribution and persistence.

Egloff *et al*.,[20] listed previous somatic pain experience (priming), psychobiographic imprinting (pain proneness), and stress (action proneness) as the key to an enhanced centralized pain response. It has become increasingly evident that muscle hyperalgesia, referred pain, referred hyperalgesia, and widespread hyperalgesia play an important role in chronic pain. Central sensitization may thus play a vital role in the persistence, amplification, and spread of pain.[21]

#### Evidence for physical therapy treatments for central sensitization:

*Patient education* Chronic pain patients mostly develop ‘pain behavior’ and treatments not addressed at their knowledge, attitudes, beliefs, and past experiences about pain are likely to be deleterious and very harmful. As any change in behavior originates from acquisition of knowledge, cognitive strategies such as education about pain works a large effect in these situations. Moseley proposed a pain neuromatrix approach[22] to management of patients with chronic pain. A series of high-quality randomized controlled trials[23–26] found that pain physiology education (PPE) reduced pain intensity and behavior in chronic low back pain patients. However, health professionals have their own difficulty in understanding the neurophysiology and neuromatrix of pain and hence avoid its application since they expect the same from their patients.[27]

*Pain-relieving modalities* such as transcutaneous electrical nerve stimulation-TENS (low frequency) through their operating ‘placebo effect’ would definitely facilitate descending inhibitory control over pain. There is a growing body of evidence for TENS in chronic pain states such as phantom limb and stump pain,[28] chronic pain conditions[29] and chronic low back pain.[30] Similar evidence demonstrated effectiveness of neuromuscular electrical stimulation in patients with post-stroke shoulder pain.[31]

*Relaxation* as a treatment technique provides an excellent adjunct to other physical therapy treatments in chronic pain states, not only to manage stress and anxiety, and their deleterious effects on the sympathetic nervous system but also to induce increases in levels of endogenous opioids—endorphins and enkephalins.[32] It operates through the central disinhibitory mechanism for relieving central sensitization. Relaxation is always the first therapeutic step in behavioral training where it reinforces acceptance and adaptive coping for pain.[33]

*Peripheral desensitization techniques* help patients such as those with hyperalgesia and allodynia. Areas with hypersensitivity to light touch can be desensitized with deep pressure since the receptors and the afferent pathway for both are entirely different.[22] Desensitization techniques can either be at the same painful region or in areas remotely away. A patient, who complains of hypersensitivity in the little finger (C_8_, T_1_ dermatome), can be desensitized by application of cutaneous deep pressure C_7_-T_1_ spinal region (related spinal level).

*Biofeedback* facilitates closed-loop motor learning to relearn normal painless movement patterns and functional activities whilst inhibiting the features of central sensitization locally in that painful part.[32]

*Guided imagery/ mental imagery/ motor imagery*[22] Utilizes cognitive-perceptual areas of the cerebral cortex to understand the planning, programming and execution of normal motor behavior and/or functional movements. Once the patient develops a mental picture of painless/ normal movement, is better able to perform the movements without causing undue pain.

*Mirror therapy*[34–36] Is very useful in extremity complex regional pain syndromes and phantom limb pain where direct visualization (not mental) of normal functioning in a painful or affected area helps the patient develop inhibition of pain through a proposed mechanism of visually induced analgesia[37] which involved a mere looking at the painful part producing pain relief.

*Virtual reality*[38] Through its dramatic visualizations involving changes in body part and/or of the environment produces not only distraction from pain but also enhances cortical mechanism of pain inhibition.

*Manual therapy* Through stimulation of wide dynamic range afferent neurons produces secondary hyperalgesia by diffuse noxious inhibitory control mechanism. The manual techniques also evoke changes in cortical pain representation and enhance the brain’s representation of body areas.[19,39,40]

### ii. Peripheral sensitization/peripheral neuropathic/ peripheral neurogenic mechanism

The International Association for the Study of Pain (IASP) defined peripheral neuropathic pain as ‘pain arising from or caused by dysfunction of peripheral nervous system’.[41] This broad anatomical definition was replaced by a more detailed functional definition provided by IASP’s Neuropathic pain Special Interest group as ‘pain arising as a direct consequence of a lesion or disease affecting the somatosensory system’.[42] Mechanical and chemical irritation of peripheral nerves can lead to musculoskeletal neural tissue injury. Repetitive compressive, tensile, friction, and vibration forces acting near anatomically narrow tissue spaces through which the neural structures pass, can cause mechanical irritation. Similarly, the injured somatic tissues adjacent to nerve structures release inflammatory substances that can chemically irritate neural tissues.[43]

Pathophysiological and pathomechanical responses to nerve injury affect the vascular, connective tissue, and impulse-conducting tissue components of the nervous system and lead to the neurobiological mechanisms responsible for the positive and negative symptoms associated with musculoskeletal peripheral neuropathic pain.[44]

#### Clinical features of peripheral neuropathic pain

Nerve trunk pain typically presents as pain or abnormal sensations along the course of the peripheral nerve that can be clinically tested using the concept of neurodynamics. Neurodynamics is a concept based on the close interaction of the mechanics and physiology of the nervous system which is to be considered while assessing and treating patients via nervous system mobilization and manual therapy.[45]

Reproduction of a patient’s pain during neurodynamic testing and presence of mechanical allodynia on nerve trunk palpation were key diagnostic signs of neural tissue mechanosensitivity.[45,46]

#### Evidence for physical therapy treatments for peripheral neuropathic pain mechanism

A recent systematic review[47] of neurodynamic mobilization as a treatment concluded overall in favor of the techniques. Neurodynamic mobilization comprising nerve sliders and tensioners, and nerve massage was shown to cause neurophysiological effects and was beneficial in relieving neuropathic pain in a variety of peripheral neural entrapment syndromes of the extremities. Nerve massage as a useful therapeutic adjunct was also shown to be beneficial for improving the sensory function of peripheral nerves.[48]

Hanai[49] found that peripheral nerve electrical stimulation inhibited pain at the spinal cord level and the author opined it was a useful therapeutic technique in neuropathic pain.

### iii. Nociceptive/ peripheral nociceptive mechanism

This mechanism was the commonly recognized one among physiotherapists[10,11] and it operates from all innervated tissues other than peripheral nerves. The pain arising from bodily tissues (both somatic and visceral) is carried by small-diameter afferent fibers and the pain transmission and perception along this mechanism was described earlier by Melzack and Wall[50] in their gate-control theory. Venugopal and Swamy[51] described three dominant mechanisms occurring in nociceptive pain—transduction, transmission and peripheral modulation.

#### Clinical features of nociceptive pain mechanism

The distribution of pain follows a characteristic anatomical pattern that corresponds to the tissue-at-fault. The pain is accompanied with other signs of inflammation such as tenderness, warmth, swelling and loss of function in the acute phases of injury. Specific aggravating and relieving factors which may be postures and/or movements will provoke symptoms. On testing active movements, passive movements and resisted isometric tests, a physical therapist can diagnose and differentiate injuries of contractile (muscle and tendon) tissues from those of inert (osseocapsuloligamentous) tissues. Manual palpation of the tissue provokes pain exhibiting as local tenderness that can be measured using pressure pain algometry.

#### Treatments for nociceptive mechanism-induced pain

Treatments for nociceptive mechanism-induced pain may include different modalities as well as conditioning exercises for healing of injury and inflammation. Modalities like heat, cold, therapeutic ultrasound, TENS and interferential current; and conditioning exercises that include stretching and flexibility exercises, massage, myofascial release and joint mobilization are also very useful for relieving pain.[3]

Manual therapy was found to be effective for symptom relief in nociceptive/musculoskeletal pain in two reviews.[52,53]

Cryotherapy or cold therapy increases pain thresholds and pain tolerance at the applied area which when combined with reduction in sensory nerve conduction velocity provides adequate pain relief.[54]

Feine *et al*.,[55] in their review found that cold therapy was useful for post-surgical pain and exercises that improve physical fitness were effective in chronic pain patients in their functional restoration.[55] Rakel and Barr[56] and Herbert *et al*.,[57] found that studies supported therapeutic exercise to be a useful modality for patients with chronic pain compared to other physical modalities like massage or electrotherapy.

### iv. Sympathetically maintained pain/ sympathetically dependent pain mechanism

The hallmark of sympathetically maintained pain (SMP) is ongoing pain and allodynia, which is typically out of proportion to the injury.[58] The causative event can vary widely from trivial injury to major trauma, or there may be no significant trauma at all. Allodynia and spontaneous ongoing pain in SMP was a result of chronic maladaptive sensitization of wide dynamic range neurons in the dorsal horn of the spinal cord, that leads to release of catecholamines and was not due to heightened sympathetic tone.[59]

#### Clinical features of sympathetically maintained pain mechanism

Symptoms of deep boring pain with altered intolerable sensations (lightning, excruciating, shock-like symptoms) mostly independent of positions and/or movements are a characteristic of SMP. Presence of autonomic signs like alterations in sweating, vasomotor function, thermoregulatory function, tissue permeability, skin trophic changes are a hallmark of diagnosis for SMP and complex regional pain syndromes. Diagnostic confirmation is only by means of a sympathetic block or sympathectomy, which mostly relieves symptoms of SMP.[60]

#### Evidence for physical therapy treatments of sympathetically maintained pain mechanism

*Thermal therapy* can be used depending upon the local temperature of the affected region and thermal sensitivity. Local warmth may indicate cold therapy and vice versa. Use of thermal modalities like heat and cold are always done as an adjunct before beginning other therapy in patients with SMP.[61]

*TENS* has an inhibitory effect on the sympathetic nervous system so has been found effective in sympathetically maintained pain.[62]

*Sympathetic slump mobilization* is a neurodynamic technique under manual therapy that addresses neural tissues, especially the sympathetic trunk.[62] The technique was shown to produce increased vasomotor and sudomotor effects (sympathetic desensitization) in upper[63] and lower extremities[64] and was shown to be a useful therapeutic technique in the treatment of patients with complex regional pain syndrome.[65]

*Exercises* can be given as a progression that includes passive range of motion, and isometric strengthening improves joint mobility and muscle strength, thereby providing alternate mechanical stimuli and graded exposure.

### v. Cognitive-affective mechanism

Casey *et al*.,[66] demonstrated that during a subtle shift from acute to chronic pain (both in duration and in mechanisms), the role of cognitive and affective factors becomes more significant, together with the inciting trauma. Psychological factors play a significant role in the causative, receptive, perceptive, cognitive, reportive and behavioral aspects of chronic pain experience among patients.[67] While cognitive factors include patients’ knowledge and maladaptive understanding of pain and ‘pain behavior’, affective factors involve emotions and feelings associated with the ‘pain experience.’

#### Evidence for physical therapy treatments for cognitive-affective pain mechanism

*Patients’ education and cognitive strategies:* (see the description under central sensitization). Education facilitates positive beliefs, problem-solving, enhanced patient functioning and self-management, and encourages active coping[68] with chronic pain. Educational interventions were shown to be equally beneficial when provided as in group therapy versus an individualized patient-therapist interactive ‘pain talk’. Such cognitive strategies have direct beneficial effects on the attention and emotions involved with the ‘pain experience’.[69] Catastrophizing thoughts can be eliminated by enhanced self-efficacy through development of adequate coping strategies and self-monitored graded return-to-activity programs.[70]

*Relaxation techniques* (refer to central sensitization mechanism)

*Hypnosis and operant conditioning* usually provided by clinical psychologists help in inducing positive behavioral changes.[71]

*Distraction* with videos or music helps reduce anxiety and depression associated with chronic pain.[72]

Morley *et al*.,[73] in their systematic review of 25 randomized controlled trials found that in comparison with alternative active treatments, cognitive-behavioral treatments produced greater beneficial changes in pain experience, cognitive coping and appraisal, and reduced behavioral expression of pain. Though cognitive-behavioral therapy (CBT) was a part of psychological therapies,[71] numerous studies published on CBT involved physical therapists[74] as intervention providers that showed large therapeutic effects on cognitive-affective outcomes. Graded exposure training,[75] graded activity with problem-solving training,[76] graded motor imagery[22] and activity pacing can be very well prescribed by physical therapists.

## DISCUSSION

This clinical commentary is the first of its kind to throw light on mechanism-based classification of pain with a physical therapy management perspective. Earlier studies utilized ‘pathomechanism-based classification (PMBC) system’ for low back-related neural leg pain which was proposed by Schafer *et al*.,[77] and studied later by many physical therapists for inter-rater reliability[78] and discriminant validity.[79] The PMBC included only four of the MBC subgroups, the cognitive-affective mechanism was not considered and hence such patients were excluded. The wide applicability of the PMBC approach to all pain conditions is questionable since it is impossible to separate a cognitive-affective mechanism from a patient’s pain experience. This paves the way for the MBC approach to be clinically more useful and widely recommended in a variety of pain conditions.

One of the mainstays in non-pharmacological interventions in pain and palliative care is physical therapy. Jones *et al*.,[80] described the importance and process of integrating a biopsychosocial model into the clinical practice of physical therapy and the highest level of evidence supports multidisciplinary biopsychosocial interventions for pain conditions such as fibromyalgia,[81] work-related neck and shoulder pain,[82] and work-related sub-acute low back pain.[83] MBC is definitely not against the biopsychosocial model though it appears often so on first look. It is imperative that central sensitization, peripheral neurogenic and nociceptive are indicative of biological dimension; central sensitization and cognitive-affective are indicative of psychological dimension; and, cognitive-affective mechanism involves social dimension. Such a biopsychosocial understanding of the mechanism-based classification of pain is essential for the successful management of pain in palliative care physical therapy practice.

Patient-centered care should focus on individualizing treatments based on mechanisms and not on diagnosis.[84] Such an evidence-informed paradigm shift[85] is necessary for physical therapists to think ‘out of the box’[86] in their clinical decision-making for pain management in palliative care. Further studies on mechanism-based classification and such classification-based treatments are essential before extrapolating the evidence to physical therapy management of patients in palliative care.

Currently, there are no existent mechanism-based treatment guidelines or recommendations. The following suggestions are provided herewith, for planning future studies on MBC:

Prevalence of MBC sub-groups in various pain conditions encountered in palliative care, Mechanism-based management using other treatments, and Effectiveness of educational and/or training programs on MBC.

“An evidence-based approach to pain management is not always possible or beneficial to the patient. In the face of inconclusive evidence, a theory-based approach may help determine if the therapeutic effect of a given physical agent has the possibility of being a useful clinical tool in the context of treating a particular patient’s mechanism of pain generation.”

-Allen.[87]

## CONCLUSION

The review was intended to provide a detailed perspective of mechanism-based classification of pain for physical therapy management in palliative care settings. However, the use of this classification will have to be better validated in patient-based research in palliative physical therapeutic care.
